# Pathophysiological In Vitro Profile of Neuronal Differentiated Cells Derived from Niemann-Pick Disease Type C2 Patient-Specific iPSCs Carrying the *NPC2* Mutations c.58G>T/c.140G>T

**DOI:** 10.3390/ijms22084009

**Published:** 2021-04-13

**Authors:** Maik Liedtke, Christin Völkner, Alexandra V. Jürs, Franziska Peter, Michael Rabenstein, Andreas Hermann, Moritz J. Frech

**Affiliations:** 1 Translational Neurodegeneration Section “Albrecht Kossel”, Department of Neurology, University Medical Center Rostock, 18147 Rostock, Germany; Maik.Liedtke@med.uni-rostock.de (M.L.); Christin.Voelkner@med.uni-rostock.de (C.V.); alexandra.juers@gmx.de (A.V.J.); franziska.peter15@gmail.com (F.P.); m.rabenstein@uni-bonn.de (M.R.); Andreas.Hermann@med.uni-rostock.de (A.H.); 2 Center for Transdisciplinary Neurosciences Rostock (CTNR), University Medical Center Rostock, 18147 Rostock, Germany; 3 German Center for Neurodegenerative Diseases (DZNE) Rostock/Greifswald, 18147 Rostock, Germany

**Keywords:** Niemann-Pick type C2 (NP-C2), cholesterol, oxidative stress, filipin, autophagy, patch clamp, hiPSC

## Abstract

Niemann-Pick type C2 (NP-C2) disease is a rare hereditary disease caused by mutations in the *NPC2* gene. NPC2 is a small, soluble protein consisting of 151 amino acids, primarily expressed in late endosomes and lysosomes (LE/LY). Together with NPC1, a transmembrane protein found in these organelles, NPC2 accomplishes the exclusion of cholesterol; thus, both proteins are essential to maintain cellular cholesterol homeostasis. Consequently, mutations in the *NPC2* or *NPC1* gene result in pathophysiological accumulation of cholesterol and sphingolipids in LE/LY. The vast majority of Niemann-Pick type C disease patients, 95%, suffer from a mutation of *NPC1*, and only 5% display a mutation of *NPC2*. The biochemical phenotype of NP-C1 and NP-C2 appears to be indistinguishable, and both diseases share several commonalities in the clinical manifestation. Studies of the pathological mechanisms underlying NP-C2 are mostly based on NP-C2 animal models and NP-C2 patient-derived fibroblasts. Recently, we established induced pluripotent stem cells (iPSCs), derived from a donor carrying the *NPC2* mutations c.58G>T/c.140G>T. Here, we present a profile of pathophysiological in vitro features, shared by NP-C1 and NP-C2, of neural differentiated cells obtained from the patient specific iPSCs. Profiling comprised a determination of the NPC2 protein level, detection of cholesterol accumulation by filipin staining, analysis of oxidative stress, and determination of autophagy. As expected, the NPC2-deficient cells displayed a significantly reduced amount of NPC2 protein, and, accordingly, we observed a significantly increased amount of cholesterol. Most notably, NPC2-deficient cells displayed only a slight increase of reactive oxygen species (ROS), suggesting that they do not suffer from oxidative stress and express catalase at a high level. As a site note, comparable NPC1-deficient cells suffer from a lack of catalase and display an increased level of ROS. In summary, this cell line provides a valuable tool to gain deeper understanding, not only of the pathogenic mechanism of NP-C2, but also of NP-C1.

## 1. Introduction

Niemann-Pick type C (NP-C) disease is a hereditary disease caused by mutations in the *NPC1* or *NPC2* gene. The proteins NPC1 and NPC2 accomplish the exclusion of cholesterol from late endosomes and lysosomes and are essential for maintaining cellular cholesterol homeostasis [1]. Consequently, mutations in *NPC1* or *NPC2* result in pathophysiological accumulation of cholesterol and sphingolipids in lysosomes and late endosomes. Comparison of clinical and biochemical phenotypes in human patients disclosed no qualitative difference, proving that NPC1 and NPC2 proteins are essential for the transport of cholesterol in lysosomal compartments in a cooperative manner [2]. Clinical manifestation of Niemann-Pick type C1 (NP-C1) disease and Niemann-Pick type C2 (NP-C2) disease share several commonalities; however, about 95% of NP-C patients show mutations of the *NPC1* gene and only 5% harbor mutations of the *NPC2* gene [3]. Naturally, the knowledge of the clinical presentation of NP-C1 is much more advances than the knowledge of NP-C2. Progression and severity of NP-C1 pathophysiology depends on the onset of the disease, wherein an early onset (early infantile form) is commonly linked to a severe phenotype with systemic involvement and an early demise of patients. An onset of NP-C1 in childhood (late infantile/juvenile form) or adulthood (adolescence/adult form) is frequently related to a neurological phenotype, although this cannot be considered as a generalized rule as the clinical presentation is heterogeneous and a clear genotype; phenotype correlation for NP-C1 is not given [3]. Regarding NP-C2, clinical case reports cite an early death of patients accompanied frequently with severe lung deficiencies [4]. Affected patients are mainly characterized by neurological dysfunction and liver damage. Neurological dysfunction causes many identifiable symptoms, including vertical supranuclear ophthalmoplegia, cataplexy, dysarthria, dysphagia, seizures, speaking and swallowing difficulty, and dementia [3]. Moreover, all of these differ greatly between patients in terms of age-of-onset, clinical presentation, disease severity, and course of the neurodegeneration. Importantly, researchers have figured out some of the genotype-phenotype correlations in NP-C patients with *NPC2* mutations.

Recently, we generated NP-C2 patient-specific induced pluripotent stem cells (iPSCs) [5]. The *NPC2* mutant cell line harbors the mutation c.58G>T/c.140G>T (p.E20X/p.C47F). The c.58G>T mutation results in a premature stop codon downstream of the signal peptide and a severely truncated protein. Patients homozygous for this mutation show a severe clinical course, with either a neonatal onset accompanied by, usually fatal, respiratory or hepatic manifestations and/or a neurological course, leading to death before the age of four [6]. Here, we report on the neuronal differentiation of these iPSCs and the evaluation of common pathophysiological features of NP-C, such as cholesterol accumulation, oxidative stress, and alterations in autophagy. Knowledge about the impact of *NPC2* mutations in neuronal cells may turn out to be important for a deeper understanding of the pathophysiological mechanisms, not only in NP-C2, but also in NP-C1.

## 2. Results

### 2.1. Differentiation of NPC2-Deficient Neurons and Glia Cells

**Figure 1:**
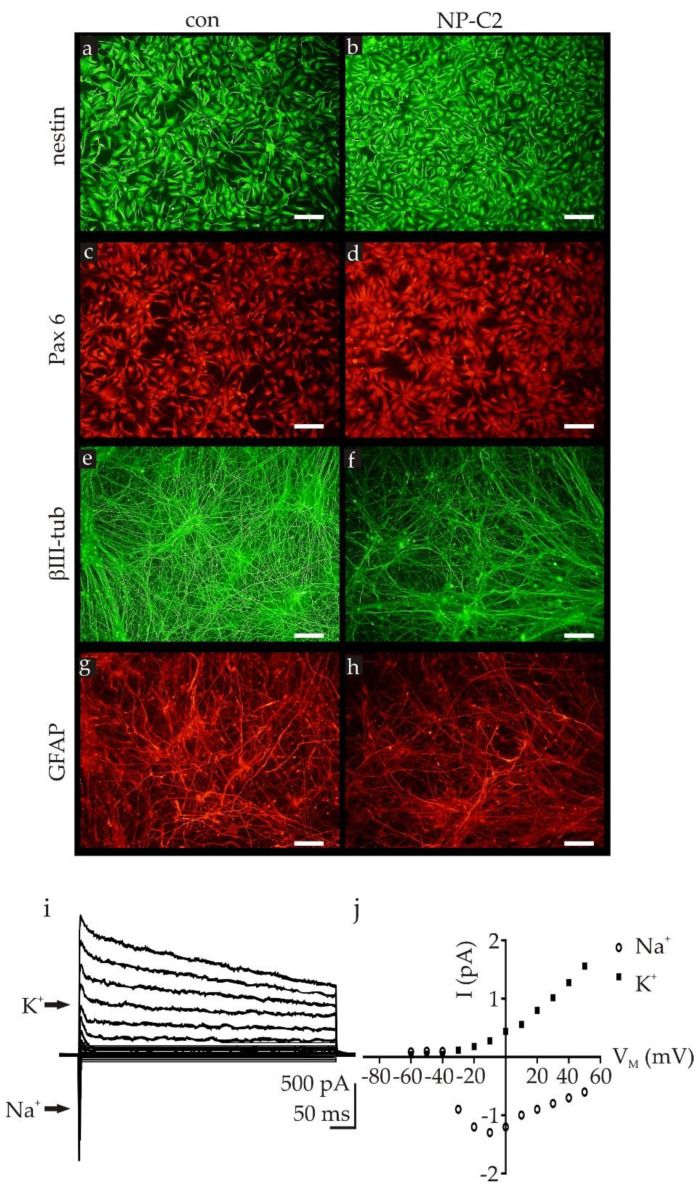
Neuronal differentiation of induced pluripotent stem cells (iPSCs). Neural progenitor cells derived from iPSCs, expressed the progenitor cell markers nestin (**a**,**b**) and Pax6 (**c**,**d**), proven by immunocytochemical staining. Neural progenitor cells (NPCs) were terminally differentiated into cultures, containing neurons and glia cells, demonstrated by immunocytochemical staining for the neuronal marker βIII-tubulin (**e**,**f**, green) and the glia cell marker GFAP (**g**,**h**, red). Neurons expressed voltage-activated Na^+^- and K^+^-channels, demonstrated by patch clamp recordings of visually identified neurons. Patch clamp measurements revealed typical inward directed Na^+^- and outward directed K^+^-currents (**i**), indicated by arrows) and (**j**) current–voltage relationship of observed Na^+^- and K^+^-currents shown in (**i**). con = control cell line; NP-C2 = NPC2-deficient cell line; Na^+^ = Na^+^-currents; K^+^ = K^+^-currents. Scale bars in a–h = 100 µm.

Recently, we developed neuronal cell models based on NP-C1-patient specific iPSCs [7,8,9]. The protocol used for this purpose contains a pre-differentiation of iPSCs into neural progenitor cells (NPCs) and a final step for differentiation of NPCs into neural differentiated cells (NDCs), namely neurons and glia cells. This protocol was used to differentiate the NPC2-deficient iPSCs into NPCs in a first step. NPC2-deficient NPCs were proven to be positive for the progenitor cell markers nestin (Figure 1a,b) and Pax6 (Figure 1c,d). Terminal differentiation of the NPC2-deficient NPCs resulted in a mixed culture of neurons, positive for βIII-tubulin (Figure 1e,f, green) and glia cells, positive for glial fibrillary acidic protein (GFAP) (Figure 1g,h, red). The differentiation into functional neurons was demonstrated by patch clamp recordings, showing the expression of voltage dependent Na^+^- and K^+^-channels (Figure 1i,j). In summary, neural differentiation of NPC2-deficient cells and control cells results in mixed cultures, containing neurons and glia cells, comparable to recently described NPC1-deficient cell model systems based on patient-specific iPSCs [7,10].

### 2.2. NPC2 Protein Level and Cholesterol Accumulation in NPC2-Deficient NDCs

**Figure 2:**
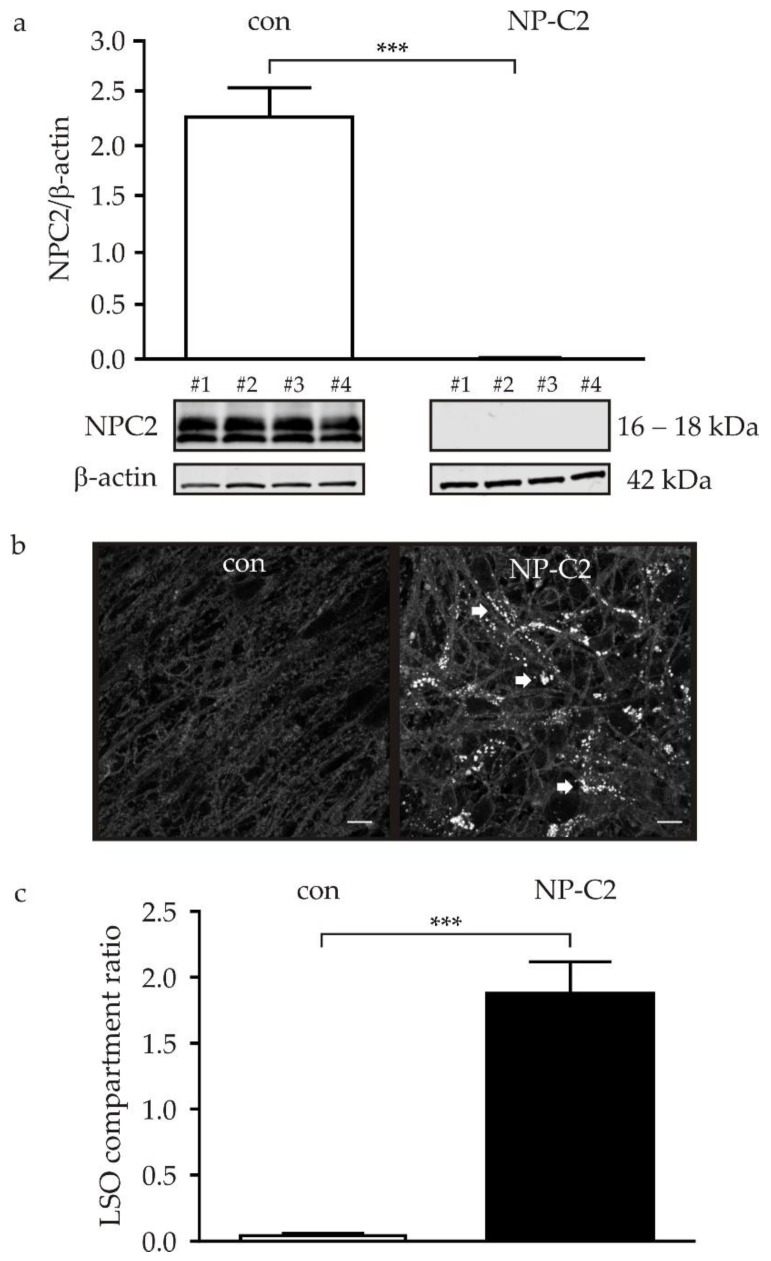
Detection of NPC2 protein and cholesterol accumulation. (**a**) Evaluation of NPC2 protein amount in control cells (white bar) and NPC2-deficient cells (black bar) by Western blot. NPC2 was detected in control cells as two distinct bands of 16 and 18 kDa. In contrast, no signal was observed in NPC2-deficient cells. An example of a complete Western blot membrane is shown in Figure S1. (**b**) Cholesterol accumulation was analyzed by means of filipin staining. In control cells (con), no obvious cholesterol accumulation was observed. In contrast, NPC2-deficient cells displayed a clearly visible accumulation (indicated by arrows). (**c**) Quantification of the lysosome-like storage organelles compartment ratio (LSO compartment ratio) revealed a significantly increased ratio in NPC2-deficient cells. *** = *p* < 0.001. con = control cell line; NPC2 = NPC2-deficient cell line. Scale bar in b = 10 µm.

Regarding the analysis of pathophysiological hallmarks of NP-C2, we firstly analyzed the amount of NPC2 protein in NDCs of the control cell line and the NPC2-deficient cell line by means of a Western blot. Control cells demonstrated two distinct bands located between 16 and 18 kDa, displaying, most likely, two isoforms of the NPC2 protein (Figure 2a). In contrast, no signal different from the background was detected in the NPC2-deficient cells, indicating a complete lack of NPC2 protein (Figure 2a). As NPC2 functions as a cholesterol transporter, shuttling cholesterol in a cooperative manner, together with NPC1, over the membrane of lysosomal compartments [1], lack of the protein should result in accumulation of cholesterol. Accordingly, the detection of cholesterol by filipin staining [11] demonstrated no obvious accumulation of cholesterol in NDCs of the control cell line (Figure 2b, left panel). In contrast, accumulation of cholesterol was perceived in cells of the NPC2-deficient cell line (Figure 2b, right panel). A quantification of the fluorescence intensity of filipin in lysosome-like storage organelles (LSO) [12] demonstrated a significantly increased amount of cholesterol in NPC2-deficient cells in contrast to the control cell line (Figure 2c).

### 2.3. Determination of ROS and Proteins of the Oxidative Stress Defense System

Oxidative stress (OS) is a common pathophysiological feature of neurodegenerative diseases, characterized by an elevated level of reactive oxygen species (ROS) and alterations of the cellular antioxidative defense system [13]. Recently, we described that NPC1-deficient cells suffer from OS, displaying elevated ROS levels and a striking reduction of catalase [14]. Here, we determined OS in the NPC2-deficient cells by measurements of ROS level, superoxide dismutase (SOD) activity, and the evaluation of mRNA and protein level of SOD1, SOD2, and catalase, representing major components of the cellular antioxidative defense system.

**Figure 3:**
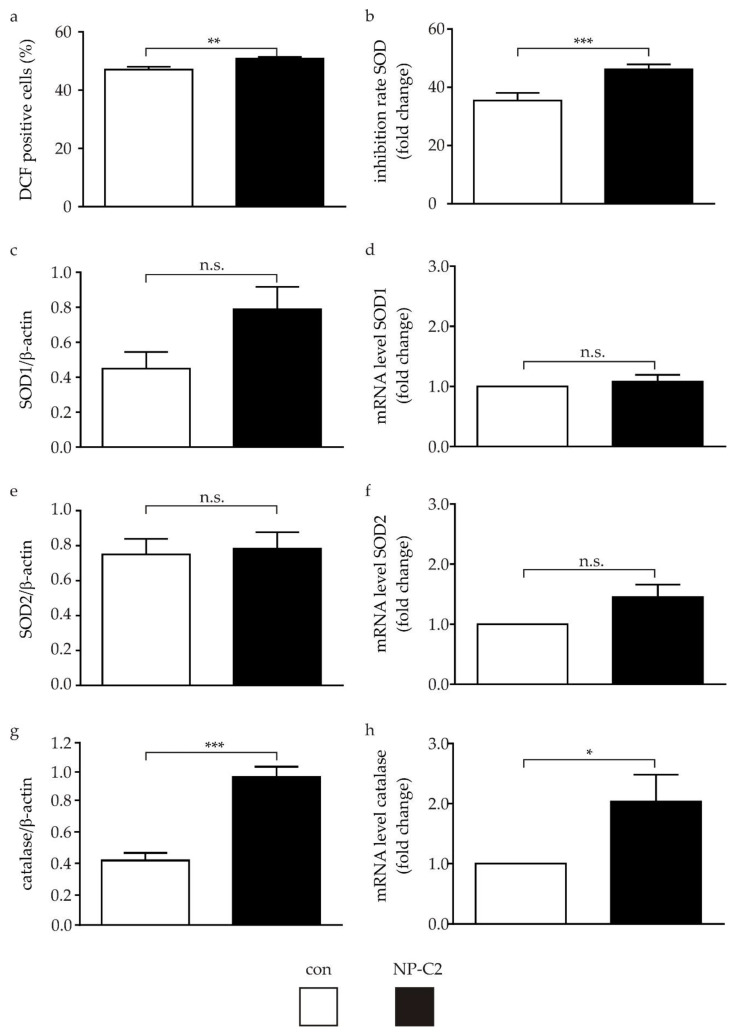
Determination of reactive oxygen species (ROS) and proteins of the antioxidative defense system. (**a**) Determination of ROS by dichlorodihydrofluorescein (DCF) assay revealed a small difference between control and NPC2-deficient cells. (**b**) Inhibition rate of SOD activity significantly increased in NPC2-deficient cells. (**c**) Analysis of the protein level of SOD1, the mRNA level for SOD1 (**d**), the protein level of SOD2 (**e**), and the mRNA level of SOD2 (**f**) were not altered in NPC2-deficient cells compared to the control cells. (**g**) The protein level of catalase, as well as the mRNA level (**h**), were significantly higher in the NPC2-deficient cells in comparison to the control cells. Examples of membranes for the Western blot analysis are shown in Figure S2. * = *p* < 0.05, ** = *p* < 0.01, *** = *p* < 0.001. con = control cell line; NP-C2 = NPC2-deficient cell line; n.s. = not statistically different.

Detection of ROS level by fluorescence analysis of dichlorodihydrofluorescein (DCF) revealed a nominal but significant increase of ROS (Figure 3a) and SOD activity measurement (Figure 3b). Regarding enzymes of the cellular antioxidative defense system, the protein level of SOD1 increased, although the increase was not significantly different to the protein level of control cells (Figure 3c). Concurrently, the mRNA level was not altered in comparison to the control cells (Figure 3d). The protein level of SOD2 was not different between control and NPC2-deficient cells (Figure 3e), although we observed a slightly increased, but statistically not significant, level of mRNA for SOD2 (Figure 3f). For catalase, we detected a significantly increased amount of catalase in the NPC2-deficient cells, accompanied by a significantly increased mRNA level, in comparison to the control cells.

### 2.4. Evaluation of Autophagy

**Figure 4:**
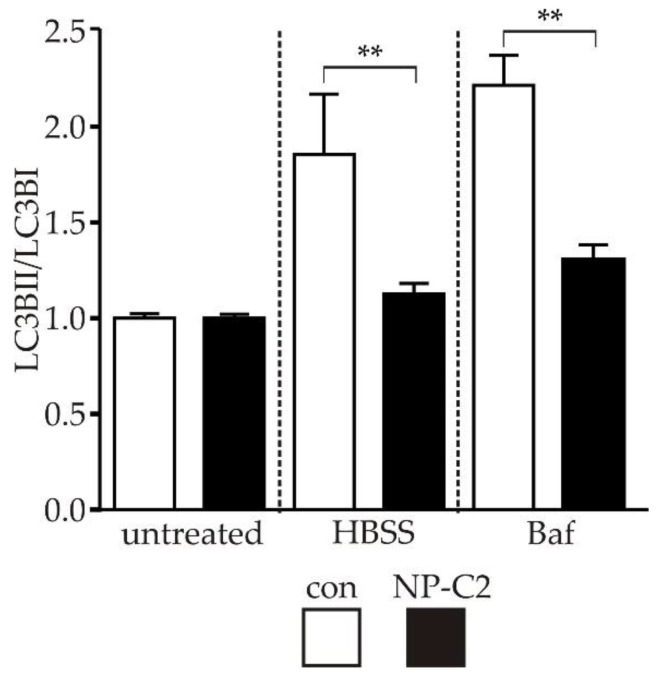
Evaluation of autophagy. To determine alterations of autophagy, the ratio of the level of LC3BII/LC3BI protein expression was analyzed by means of Western blot. Cells cultured under basal conditions (indicated as untreated) did not show a difference in the LC3BII/LC3BI ratio. Control cells cultured under condition of starvation (indicated as HBSS) showed increased LC3BII/LC3BI ratio, in contrast to NPC2-deficient cells. A comparable reaction of control cells was observed after the application of Bafilomycin A1 (Baf). Example of a complete Western blot membrane is shown in Figure S3. ** = *p* < 0.01

Disturbances in the autophagic flux are frequently reported for neurodegenerative diseases, such as Alzheimer’s disease, Parkinson’s disease, amyotrophic lateral sclerosis [15], and NP-C1 disease [16]. Here, we analyzed the autophagic flux by means of Western blot analysis of LC3BI and LC3BII, two major components of the autophagy pathway. Analysis revealed that the basal level of the LC3BII/LC3BI ratio was similar in the control cells and NPC2-deficient cells (Figure 4, indicated as untreated). However, keeping the cells under conditions of nutrient deprivation (Figure 4, indicated as HBSS), induced a significantly increased ratio of LC3BII/LC3BI in control cells, but not in NPC2-deficient cells. Comparable results were obtained for the treatment of the cells with Bafilomycin A1, a blocker of V-ATPase. Treatment induced a significantly higher LC3BII/LC3BI ratio in control cells in comparison to NPC2-deficient cells (Figure 4, indicated as Baf).

## 3. Discussion

Niemann-Pick type C2 disease is caused by mutations in the *NPC2* gene, which is mapped on the q-arm of chromosome 14 at position 24.3 in humans. *NPC2* is 13.5 kb long and composed of five exons, encoding for a protein previously known as HE1, a major secretory protein in the human epididymis [17]. The NPC2 protein is a small, soluble, ubiquitously expressed, lysosomal glycoprotein that specifically binds unesterified cholesterol with a submicromolar affinity at neutral and acidic pH [3]. NPC2 and NPC1 proteins are required for proper transport of cholesterol, and thus, mutations in either gene lead to defects in cholesterol export from lysosomes, resulting in the typical accumulation of cholesterol observed in NPC2- and NPC1-deficient cells [3]. As only 5% of patients suffering from Niemann-Pick disease harbor a mutation in *NPC2* and 95% harbor a mutation in *NPC1*, the knowledge regarding the pathophysiology of NP-C1 is much more extensive than that regarding NP-C2. However, regarding the biochemical phenotype, NP-C2- and NP-C1-patients cannot be distinguished by biochemical analysis, for example, by the mentioned cholesterol accumulation. Currently, more than 20 different mutations of *NPC2* have been described, reported in about 50 clinical cases, wherein the majority of mutations are severe, regularly resulting in early disease onset with a short lifespan [18].

The fibroblasts we used, and consequently the derived iPSCs and their derivatives, harbor the compound heterozygous mutation c.58G>T/c.140G>T. The c.58G>T mutation appears to be frequent in NP-C2 patients, as far as one can speak of frequent in view of the small number of known NP-C2 patients. Recently, Dardis and colleagues [19] described the molecular genetics of an NP-C patient cohort, comprising 105 patients. As to be expected, the majority of the patients carried *NPC1* mutations and only eight patients suffered from mutations in the *NPC2* gene. Out of these eight patients, two patients were homozygous for the c.58G>T mutation. An assessment of pathophysiological features on a cellular level can be done with patient-derived fibroblasts, but it is hard to analyze cells, such as neurons or hepatocytes, as it is almost impossible to access these cell types by biopsies from patients. However, these cell types are of special interest, as NP-C patients display neurological symptoms and the majority suffer from hepatosplenomegaly [3]. Certainly, iPSC-derived cells offer a unique access to such an analysis. In this work, we investigated pathophysiological features of iPSC-derived neuronal cells, as recently described in a comparable manner, for iPSC-based cell models for NP-C1 [20]. The neuronal differentiation of NPC2-deficient iPSCs resulted in a mixed culture of neurons and glia cells, comparable to results obtained from NPC1-deficient cells, without any obvious differences in the morphology or function of the NPC2-deficient cells. However, we have not yet undertaken an in depth functional characterization of these cells, and it is unclear whether NPC2-deficient neurons display comparable functional deficits, such as NPC1-deficient cells, which we described recently [10]. As a first step, we did a basic characterization of the biochemical phenotype, comprising an analysis of cholesterol accumulation, signs of oxidative stress, and alterations of autophagy, which has been explored numerously in NPC1-deficient cells (for review, refer to [20]).

### 3.1. Lipid Accumulation

NP-C2 and NP-C1 are characterized by the accumulation of cholesterol and sphingolipids in late endosomes and lysosomes (LE/LY) due to the absence of functional NPC2 protein. Here, we demonstrated a clear reduction of the NPC2 protein in iPSC-derived cells by Western blot (Figure 2a), accompanied by accumulation of cholesterol, shown by filipin staining (Figure 2b). Cholesterol accumulation was quantified by the analysis of the LSO compartment ratio [12], demonstrating an increased ratio and thus an accumulation of cholesterol in NPC2-deficient cells. These results are in accordance to results we obtained from NPC1-deficient cells derived from NP-C1-patient-specific iPSCs [8]. Filipin staining can be used as a tool for clinical diagnosis in patient-derived fibroblasts, both from NP-C2 and NP-C1 patients. However, at present, biomarkers or genetic analyses are used for the diagnosis of NP-C [21]. These are more reliable because filipin staining may fail as a diagnostic tool in patients with a mild clinical phenotype or in patients with a biochemical variant phenotype [22,23]. In these patients, cholesterol accumulation cannot be detected, despite clinical symptoms [24]. To which extend this can be observed for NP-C2 by in vitro cell models stays elusive as long as no further human iPSC-based model systems for NP-C2 are available. The above-mentioned Italian cohort of 105 NP-C patients comprised eight NP-C2 patients, wherein two patients were homozygous for the c.58G>T mutation. Unfortunately, a filipin staining of fibroblasts of these patients is not available.

### 3.2. Oxidative Stress in NPC2-Deficient Cells

Oxidative stress (OS) is a pathophysiological feature commonly observed in neurodegenerative diseases [13]. OS is described for various NPC1-deficient cells (for review, refer to [20]), including NPC1-deficient cells derived from patient-specific iPSCs. Thus, we were interested to see if NPC2-deficient cells, derived from iPSCs, would display hallmarks of OS. Therefore, we determined OS by the measurement of the ROS level, SOD activity, and the evaluation of mRNA and protein levels of SOD1, SOD2, and catalase, representing major components of the cellular antioxidative defense system. Surprisingly, we could not find clear signs of oxidative stress, as we have shown in NPC1-deficient cells [14]. A possible explanation for the low ROS values could be a counteraction of the cells against the existing OS by upregulation of the antioxidative defense system. The increased SOD1 protein level and SOD2 mRNA level, although not significant, may lead to an elevated SOD activity, which was observed in the SOD activity assay. SOD, as a key enzyme of the antioxidant defense system, converts superoxide anions into hydrogen peroxide and molecular oxygen [25], which are eliminated by different enzymes of the cell, particularly the catalase [26]. The catalase level observed here supports this assumption, although this finding is in clear contrast to our findings in NPC1-deficient cells, where we observed a striking reduction in the catalase level. This suggests that NPC1-deficient cells suffer from OS because they lack catalase. Conversely, it can be concluded that NPC2-deficient cells, having no deficiency in catalase, do not suffer from OS. Certainly, further studies on NPC2-deficient cells are needed to clarify whether this represents a general difference between NPC1 and NPC2 or rather a mutation-specific effect. Unfortunately, a comparison with other studies is currently not possible, as no studies are available for NPC2-deficient cells regarding OS. Furthermore, it would be interesting to know whether NPC1- and NPC2-deficient cells show differences in DNA methylation. It has been shown that exposition to ROS impacts the epigenetic methylation of the promotor region of catalase [27]. Thus, one might speculate that the epigenetic modulation of NPC1 and NPC2 differs, just as the exposure to ROS differs. Another interesting aspect could be the expression of NF-E2-related factor 2 (Nrf-2). An increased expression of Nrf-2 was shown in NPC2-deficient CHO cells [28]. Nrf-2 is not only an important key player upstream of catalase, SOD1 and SOD2 [29,30], but also displays a link to autophagy. Increased expression of sequestosome 1 (SQSTM1)/p62, which plays an important role in autophagy, can lead to increased expression of Nrf-2, which in turn can lead to an increased response of the anti-oxidative defense system and thus can counteract an increased occurrence of ROS. The extent to which such a mechanism plays a role in the NPC2-deficient iPSC-derived cells studied here will be the subject of further investigation, as will analyses of the NPC1-deficient iPSC-derived cell models to uncover possible differences between NP-C2 and NP-C1.

### 3.3. Autophagy in NPC2-Deficient Cells

Macroautophagy, also referred to as autophagy, is a physiological process to recycle proteins and organelles and maintain cellular energy and nutrient homeostasis under starvation [31]. Through the autophagic pathway cells can eliminate dysfunctional cellular components and, consequently, defects in autophagy can lead to an accumulation of such components. Disruptions in the autophagic pathway are commonly observed in neurodegenerative diseases, such as Alzheimer’s disease [32], Parkinson’s disease [33], amyotrophic lateral sclerosis [34], and Niemann-Pick type C disease [16]. Autophagy is a multistep process with the mammalian target of rapamycin complex 1 (mTORC1) as one of the main regulatory elements [35]. Under nutrient deprivation or cellular stress, autophagy is upregulated by the inhibition of the mTORC1 complex. Subsequently, the formation of autophagosomes, via formation of the Unc-51-like kinase 1 (ULK1) complex, is initiated. During vesicle formation, the material that should be degraded is engulfed in these autophagosomes, which in later steps fuse with lysosomes, forming so-called autolysosomes, where the engulfed material is finally degraded [36].

In NP-C1, disturbances of autophagy are described for several model systems [37,38,39], but the contribution to the pathophysiology of NP-C1 is not yet fully understood. However, recently, it was shown that mutations in *NPC2* also affect autophagy. An *NPC2* knock-down in adipocytes hampered a starvation-induced activation of autophagy. Furthermore, the increase of LC3BII after treatment with Bafilomycin A1 was reduced compared to controls, indicating a defect in autophagosome formation [40]. Similar to the observed phenotype in adipocytes, we found an inhibited autophagy induction by starvation. During Bafilomycin A1 treatment, the increase of the LC3BII/LC3BI ratio, due to the block of the autophagic clearance, was significantly lower compared to the healthy control, supporting our hypothesis of an impaired autophagy induction. These results point to a significantly inhibited induction of autophagy at the step of autophagosome formation, which may lead to an accumulation of damaged organelles and proteins due to their inhibited clearance. As mentioned above, an increased level of SQSTM1/p62 could indicate defective autophagosome formation, whereas the general induction and labeling of polyubiquitinated proteins by SQSTM1/p62 could still be functional.

Additionally, it was reported that mice lacking the autophagy-related protein 7 (ATG7) are not able to upregulate the autophagosome formation under starvation [41]. The c-Jun N-terminal protein kinase 1 (JNK1) is another key player during starvation-induced autophagy. JNK1 phosphorylates the anti-apoptotic protein Bcl2, mediating its dissociation from Beclin-1, which, in the end, induces autophagosome formation [42], making it a reasonable target for further evaluation, besides ATG7. However, we can only speculate whether the above mechanisms operate in the cell model used here, and a more detailed analysis of the induction of autophagosome formation and the proteins involved should be the subject of further studies.

Our findings in the NPC2-deficient cells differ from results observed in NPC1-deficient cells. In the latter, an increased LC3BII/LC3BI ratio and also increased levels of SQSTM1/p62 [43], most likely due to an accumulation of autophagic vesicles, is reported [37,38,44]. During nutrient deprivation, cells carrying an *NPC1* mutation are capable of increasing the autophagosome production and thus enhancing the recycling of damaged organelles and proteins [45]. After blockage of the autophagosome-lysosome fusion by Bafilomycin A1, no differences between NPC1-deficient cells and healthy control cells could be detected, suggesting that the clearance of autophagic vesicles is impacted in a later step of the autophagic pathway [46]. In conclusion, despite the characteristic cholesterol accumulation, NP-C2 differs from NP-C1 in regard to an impacted anti-oxidative defense system and the defects observed in the autophagic pathway. This points towards other functions of the NPC1 and NPC2 protein in these pathways, beside their main task of cholesterol transport.

## 4. Materials and Methods

### 4.1. Culture of Human Fibroblasts

Human dermal fibroblast cell lines from male donors were obtained from Coriell Institute for Medical Research (Camden, NJ, USA). NPC2-deficient cell line GM18455 harbored the heterozygous mutation: c.58G>T [p.E20X]; c.140G>T [p.C47F]. Cell line GM05659 was used as the control. Cells were cultured in fibroblast medium containing DMEM high glucose, 10% FBS, and 1% penicillin/streptomycin.

### 4.2. Generation and Culture of iPSCs

Human iPSCs were generated as described recently [5]. In brief, retroviruses were produced in HEK293FT cells using a retroviral vector encoding for GFP and one of the transcription factors (Sox2, Klf4, Oct4, or c-Myc), as well as VSV-G and Gag-Pol by XtremeGene 9 (Roche Diagnostics GmbH, Mannheim, Germany). The human fibroblasts were transduced with Sox2, Oct4, Klf4, and c-Myc in the presence of 5 µg/mL protamine sulfate (in fibroblast medium). After 48 h, cells were reseeded onto a gelatin-coated 6 cm-dish. The following day, the medium was replaced with iPSC medium, supplemented with 0.5 mM valproic acid to further increase the efficiency of reprogramming. The medium was changed daily, and valproic acid was omitted after 7 days. Initial iPSC colonies were picked and further cultured on 45,000 feeder cells/cm^2^ and subsequently further cultured on Matrigel (Corning, NY, USA) in mTESR1 medium (Stemcell Technologies, Vancouver, BC, Canada). The medium was changed daily and cells were passaged weekly.

### 4.3. Neural and Neuronal Differentiation

Neural differentiation of iPSCs was induced by density-dependent growing of iPSCs on Matrigel (Corning, NY, USA) to induce generation of neural rosettes. Once neural rosettes had been formed spontaneously, neural progenitor cells (NPCs) were isolated using magnetic beads against the surface marker PSA-NCAM (Miltenyi Biotec, Bergisch Gladbach, Germany). NPCs were seeded at an expansion density of 100,000 cells/cm^2^ on poly-L-ornithine (15 µg/mL; Sigma Aldrich, St. Louis, MO, USA)/laminin (10 µg/mL; Trevigen, Gaithersburg, MD, USA)-coated dishes in proliferation medium containing 40% DMEM, 60% DMEM/F-12, 1X B27, 0.5% penicillin/streptomycin, 20 ng/mL FGF2 (Amsbio, Abingdon, UK), and 20 ng/mL EGF (Peprotech, Hamburg, Germany). For terminal neuronal differentiation, cells were plated at a density of 45,000 cells/cm^2^ in differentiation medium, containing 40% DMEM, 60% DMEM/F-12, 1X B27, and 0.5% penicillin/streptomycin, which was changed every 4 days over a period of 6 weeks.

### 4.4. Patch Clamp Recordings

Patch clamp recordings were performed as recently described [10]. In brief, recordings were made in the whole cell configuration in voltage clamp mode or current clamp mode, using an EPC-10 amplifier (Heka, Lambrecht, Germany). Current clamp mode was used to apply current steps to induce action potentials or measure spontaneous action potentials. Voltage steps ranging from −60 mV to +50 mV in 10 mV increments were applied in the voltage clamp mode to elicit currents mediated by voltage gated channels (VGCs). Postsynaptic currents were measured in the voltage clamp mode at a holding potential (VH) of −60 mV. Borosilicate glass tubing (Harvard Apparatus, Holliston, MA, USA) was used to pull patch pipettes. Pipette solution contained (mM): KCl 130, NaCl 10, HEPES 10, EGTA 11, MgCl_2_ × 6H_2_O 1, CaCl_2_ × H_2_O 2, and Mg-ATP 2. pH was adjusted to 7.2. When filled, patch pipettes had a resistance of 6–8 MΩ. Cell cultures were continuously superfused with an extracellular solution consisting of (mM): NaCl 125, KCl 2.5, CaCl_2_ × H_2_O 2, MgCl_2_ × 6H_2_O 1, and NaHCO_3_ 26, NaH_2_PO_4_ × H_2_O 1.25, glucose × H_2_O 25.

### 4.5. Evaluation of the Autophagic Flux

Cells were kept in HBSS (Sigma Aldrich, St. Louis, MO, USA), supplemented with 5 mg/mL magnesium and 6.6 mg/mL calcium for 6 h to simulate nutrient starvation and induce autophagy. Bafilomycin A1 from Streptomyces griseus (Sigma Aldrich, St. Louis, MO, USA) was used at a final concentration of 50 nM for 6 h to block the clearance of autophagosomes.

### 4.6. Western Blot

Western blot was performed as recently described [14]. In brief, whole cell lysates were prepared by incubation of the cells for 30 min on ice in RIPA-lysis buffer, containing in mM: TRIS 20, NaCl 137, sodium deoxycholate 12, EDTA 2, supplemented with 0.1% SDS, 1% Triton^®^ X-100, 10% glycerol with cOmplete™, and Mini, EDTA-free Protease Inhibitor Cocktail (Roche Diagnostics GmbH, Mannheim, Germany). Lysates were centrifuged at 15,000× *g* for 25 min at 4 °C. Protein concentration of the supernatant was determined using the Pierce™ BCA Protein Assay Kit (Thermo Fisher Scientific, Waltham, MA, USA) according to the manufacturer´s instructions. Samples were boiled for 5 min at 95 °C in 5× Laemmli-buffer (125 mM TRIS, 20% glycerol, 2% SDS, 5% β-mercaptoethanol, 10% bromphenol blue) and subsequently centrifuged at 22,000× *g* for 1 min at 4 °C. For electrophoresis Criterion™, Vertical Electrophoresis Cell with Criterion™ TGX Stain-Free™ Precast Gels (4–15%) (Bio-Rad Laboratories, Hercules, CA, USA) were used. Electrophoresis buffer contained 250 mM TRIS, 2 M glycine, and 0.1% SDS. For Western blot, the Trans-Blot^®^ Turbo™ Transfer System with Trans-Bolt^®^ Turbo™ Transfer Pack (Midi Format, 0.2µm Nitrocellulose, Bio-Rad Laboratories, Hercules, CA, USA) was used. Afterwards, the membranes were washed in TRIS-buffered saline (TBS), containing 20 mM TRIS and 137 mM NaCl (pH 7.5) for 5 min and blocked with 5% BSA (Carl Roth GmbH & Co. KG, Karlsruhe, Germany) or milk in TBS supplemented with 0.1% Tween^®^ 20 (TBST) for 1 h. For protein detection of catalase, SOD1, SOD2, and LC3BII/I membranes were incubated with primary antibody solution (3% BSA in TBST) overnight at 4 °C and for 1 h at room temperature for detection of GAPDH and β-actin. For detection of NPC2 protein, membranes were incubated with primary antibody solution (3% milk in TBST) overnight at 4 °C. Subsequently, membranes were washed 3× with TBST and incubated for 1 h with DyLight™ secondary antibody. Precision Plus Protein Dual Xtra Standards (Bio-Rad Laboratories, Hercules, CA, USA) was used as a molecular weight marker. Finally, membranes were washed 3× with TBST and 1× with TBS and dried. The Odyssey Infrared Imaging System (LI-COR Biosciences GmbH, Bad Homburg, Germany) was used to visualize and quantify the protein signals. Expression of β-actin was used to normalize the expression of catalase, SOD1, SOD2, and NPC2, and the expression of GAPDH was used to normalize the expression of LC3BI and II. Used antibodies are listed in Supplementary Table S1.

### 4.7. Filipin Staining

To determine the subcellular cholesterol accumulation, NDCs were loaded with 60 µg/mL low density lipoprotein in differentiation medium (40% DMEM, 60% DMEM/F-12, 1X B27, 0.5% penicillin/streptomycin) for 24 h. For visualization of unesterified cholesterol, cells were fixed with 4% paraformaldehyde for 15 min, washed 3 × 5 min in PBS at room temperature, and incubated with 0.1 mg/mL filipin for 45 min. After three washing steps with PBS, cells were mounted with Fluoromount-G (SouthernBiotech, Birmingham, UK). Filipin fluorescence intensities were quantitatively determined by taking 10 random pictures of three replicates using a Laser Scanning Microscope 780 (Zeiss, Oberkochen, Germany). Analysis was performed using ImageJ [47] by determining the LSO compartment ratio [12].

### 4.8. Immunocytochemical Staining

Pluripotency of neural progenitor cells was proven by staining for Sox2, nestin, and Pax6. Specimens were incubated with primary antibodies overnight at 4 °C and with secondary antibodies for 1 h at room temperature in 1% BSA/PBS. Subsequently, cells were washed three times with PBS and mounted with Fluoromount-G (SouthernBiotech, Birmingham, UK). Differentiated cells were stained for the neuronal marker βIII-tubulin and the glia cell marker GFAP. Therefore, cells were permeabilized using 0.1% Triton X-100 for 5 min on ice. Cells were incubated with primary antibodies overnight at 4 °C in 1% BSA/PBS, followed by three washing steps with PBS. Secondary antibodies were added for 1 h at room temperature in 1% BSA/PBS. After washing with PBS, cells were stained with DAPI (5 min, 250 ng/mL), washed three times, and mounted with Fluoromount-G^®^ (SouthernBiotech, Birmingham, UK). Pictures were taken with a Biozero 8000 microscope system (Keyence, Hamburg, Germany). Used primary and secondary antibodies are listed in Table S1.

### 4.9. DCF Fluorescence Measurement of ROS Level

The DCF Cellular ROS Detection Assay Kit (Abcam, Cambridge, UK) was used according to the manufacturer’s manual to determine intracellular ROS level, as described recently [14]. In brief, cells were incubated with 0.6 µM 2′,7′-dichlorodihydrofluorescein diacetate (H2DCFDA) in phenol red free media (phenol red free DMEM, 1 mM pyruvate, 1% penicillin/streptomycin) for 5 min in the dark at 37 °C. To harvest cells, cultures were washed twice with PBS and incubated with Accutase (Stemcell technologies, Cologne, Germany), and a reaction was stopped with phenol red free media after 5 min. Propidium iodide (PI, 1.5 µM) was added to the cell suspension shortly before the measurement to detect apoptotic/necrotic cells during FACS analysis. For DCF fluorescence measurement excitation, a wavelength of 485 nm was used and emission was detected at a wavelength of 535 nm. PI was excited with 493 nm and emission was measured at 585 nm. A FACSCalibur (BD, Heidelberg, Germany) in combination with CellQuest Pro software (BD, Heidelberg, Germany) was used for data sampling. Data were analyzed with FCSalyzer 0.9.18-alpha software (https://sourceforge.net/projects/fcsalyzer, accessed January 2021). PI-negative and DCF-positive cells were defined as ROS positive cells. An example of a FACS-analysis is shown in Figure S4.

### 4.10. Measurement of SOD Activity

A SOD Determination Kit (Sigma-Aldrich, St. Louis, MO, USA) was used accordingly to the manufacturer’s manual measure SOD activity, as described recently [14]. In brief, cells were lysated with SOD lysis buffer (0.1 M TRIS/HCl, 1% Triton^®^ X-100, 10% glycerol, 0.05% SDS, supplemented with cOmplete™, Mini, EDTA-free Protease Inhibitor Cocktail (Roche Diagnostics GmbH, Mannheim, Germany)) for 30 min on ice. Subsequently, cells were frozen with liquid nitrogen and thawed 5 times at room temperature and subjected to 10 ultrasonic pulses (560 W, 1 Hz). Afterwards, samples were centrifuged at 15,000× *g* for 25 min at 4 °C. The Pierce™ BCA Protein Assay Kit (Thermo Fisher Scientific, Waltham, MA, USA) was used to measure protein concentration of the supernatant. Samples were treated with or without xanthinoxidase enzyme solution, and WST-1 working solution was added. Cells were incubated for 20 min in the dark at 37 °C under agitation. Absorption was measured at 450 nm with a Spark^®^ plate reader and SparkControl Magellan software (Tecan, Männedorf, CH, Switzerland). The SOD activity was calculated as follows: SOD activity (inhibition rate %) = [(Amax − Amin) − (Asample − Abase value)]/(Amax − Amin) × 100. This value was normalized to the protein concentration of the sample.

### 4.11. RT-qPCR

Level of mRNAs were determined by quantitative real-time polymerase chain reaction (RT-qPCR), as recently described [14]. In brief, RNA was isolated using Quick-RNATM Mini-Prep Kit (Zymo Research Europe GmbH, Freiburg, Germany) and cDNA was obtained using QuantiTect Reverse Transcription Kit (Qiagen, Hilden, Germany). PCR was performed using the FastStart DNA SYBRGreen Plus Kit (Roche Diagnostics GmbH, Mannheim, Germany), according to the manufacturer’s instructions, with LightCycler Nano (Roche Diagnostics GmbH, Mannheim, Germany) in combination with LightCycler Nano 1.1 software or Rotor Gene Q (Qiagen, Hilden, Germany) and Rotor-Gene Q Series Software version 2.3.1 (Qiagen, Hilden, Germany). Protocol started with an initial denaturation at 95 °C for 600 s, followed by 40 cycles of 20 s at 95 °C for denaturation and annealing with the primer specific annealing temperature and 23 s at 72 °C for extension. Integrity of PCR products was proven with a melting curve, obtained by a step of 60 s at 65 °C and 20 s at 95 °C. The YWHAZ gene was used as a reference, and the mRNA amount in the genes of interest was normalized to YWHAZ. All samples were run in technical duplicates. Relative changes in mRNA amount were calculated using ΔCt values by means of the Pfaffl method [48]. For forward and reverse primer sequences, see Supplementary Table S1.

### 4.12. Statistical Analysis

Data are presented as mean ± SEM of at least three independent replicates. Statistical analysis was performed with GraphPad Prism 6.07 (GraphPad Software Inc., San Diego, CA, USA). The D’Agostino-Pearson normality test was used to test the data sets for normal distribution. Statistical significance was tested by using an unpaired t-test or ordinary one-way ANOVA test with Dunnett’s multiple comparisons test. *p*-values < 0.05 were considered statistically significant, with * = *p* < 0.05, * = *p* < 0.01, and *** = *p* < 0.001.

## 5. Conclusions

NP-C2 patients cannot be distinguished from NP-C1 patients based on clinical presentation. Similarly, no differences were found at the cellular level with biochemical analyses. However, the extent to which this applies to nerve or liver cells is insufficiently investigated, as there are no human cell models for this. This gap is at least partially filled by the present publication. In NPC2-deficient neuronally-differentiated cells, we were able to demonstrate typical cellular pathophysiological features that have also been described for NPC1-deficient cells. However, we observed differences, in markers of oxidative stress and autophagy, between NPC2- and NPC1-deficient cells. It would be interesting to know whether there are further differences or whether the pathophysiological expression in other affected cells, such as liver cells, also differs. For this purpose, iPSC-based model systems, such as the one described here, are ideally suited and we are convinced that further work with such model systems will contribute to a better understanding not only of the pathology of NP-C2 and NP-C1, but also of other lysosomal storage diseases.

## Data Availability

The data presented in this study are available on request from the corresponding author.

## Supplementary Materials

The following are available online at https://www.mdpi.com/article/10.3390/ijms22084009/s1.
