# Supplementary material for: Pathophysiological In Vitro Profile of Neuronal Differentiated Cells Derived from Niemann-Pick Disease Type C2 Patient-Specific iPSCs Carrying the NPC2 Mutations c.58G>T/c.140G>T
Source: Int J Mol Sci. 2021 Apr 13;22(8):4009. doi: 10.3390/ijms22084009 (PMC8069078; doi:10.3390/ijms22084009)
Supplement: Supplementary file 1 [file ijms-22-04009-s001.zip › Supplementary Figure S1.pdf]

Supplementary Figure S1

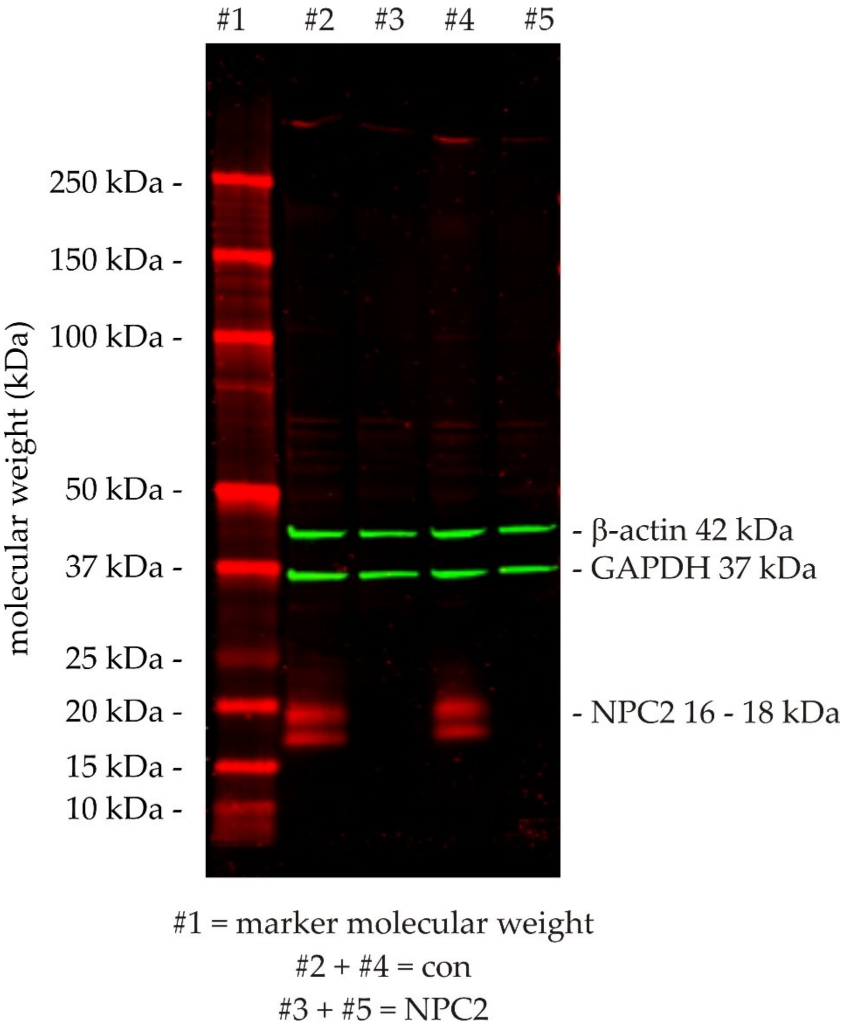

Supplementary Figure S1: Example of Western Blot to determine the amount of NPC2 protein #1 = standard marker ladder to determine molecular weight, shown in red. #2 and #4 = probes of control cells (con) showing two distinct bands of NPC2 protein in red. #3 and #5 = probes of NPC2-deficient cells (NPC2) showing no signal for NPC2. β-actin and GAPDH are shown in green.
