# Supplementary material for: Pathophysiological In Vitro Profile of Neuronal Differentiated Cells Derived from Niemann-Pick Disease Type C2 Patient-Specific iPSCs Carrying the NPC2 Mutations c.58G>T/c.140G>T
Source: Int J Mol Sci. 2021 Apr 13;22(8):4009. doi: 10.3390/ijms22084009 (PMC8069078; doi:10.3390/ijms22084009)
Supplement: Supplementary file 1 [file ijms-22-04009-s001.zip › Supplementary Figure S2.pdf]

Supplementary Figure S2

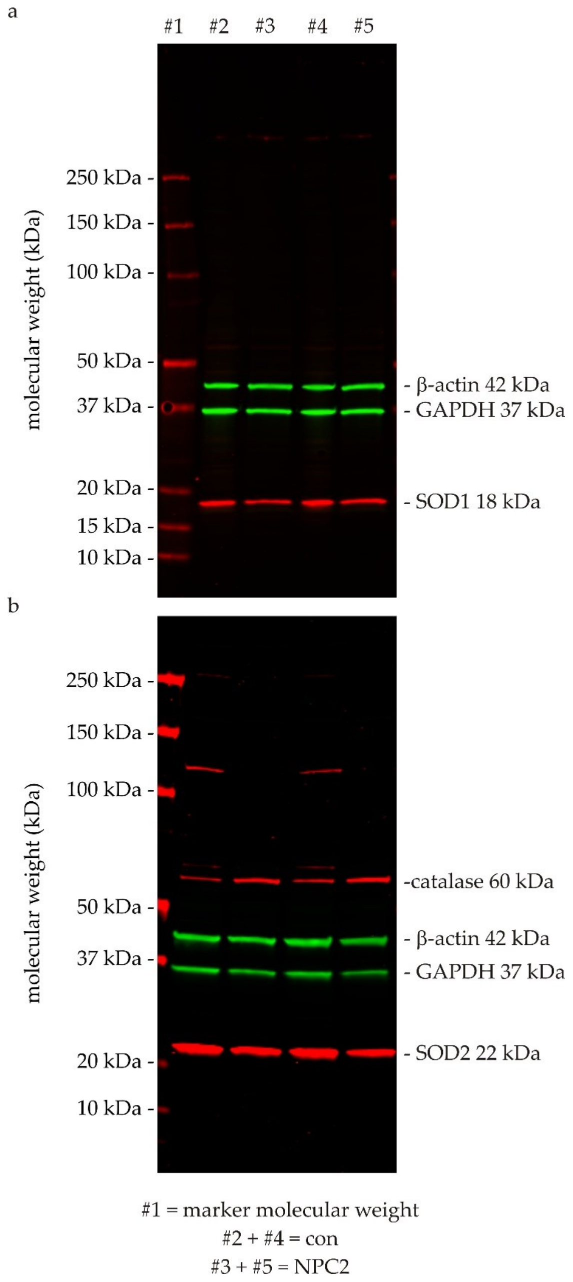

Supplementary Figure S2: Example of Western Blot to determine the amount of SOD1, SOD2 and catalase protein

(a) #1 = standard marker ladder to determine molecular weight, shown in red. #2 and #4 = probes of control cells (con) showing bands of SOD1 protein, shown in red. #3 and #5 = probes of NPC2-deficient cells (NPC2) showing SOD1 protein in red. β-actin and GAPDH are shown in green. (b) #1 = standard marker ladder to determine molecular weight, shown in red. #2 and #4 = probes of control cells (con) showing bands of catalase and SOD2 protein in red. #3 and #5 = probes of NPC2-deficient cells (NPC2) catalase and SOD2 protein, both shown in red. β-actin and GAPDH are shown in green.
