# Supplementary material for: Pathophysiological In Vitro Profile of Neuronal Differentiated Cells Derived from Niemann-Pick Disease Type C2 Patient-Specific iPSCs Carrying the NPC2 Mutations c.58G>T/c.140G>T
Source: Int J Mol Sci. 2021 Apr 13;22(8):4009. doi: 10.3390/ijms22084009 (PMC8069078; doi:10.3390/ijms22084009)
Supplement: Supplementary file 1 [file ijms-22-04009-s001.zip › Supplementary Figure S3.pdf]

Supplementary Figure S3

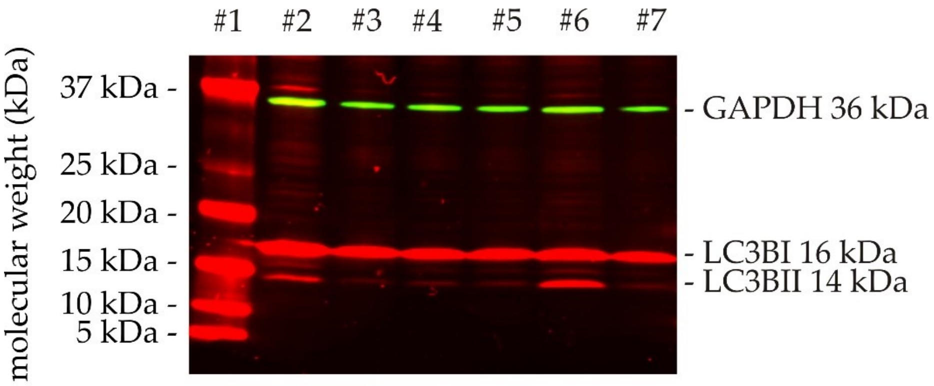

#1 = marker molecular weight
#2 = con untreated
#3 = NPC2 untreated
#4 = con HBSS
#5 = NPC2 HBSS
#6 = con Baf
#7 = NPC2 Baf

Supplementary Figure S3: Example of Western Blot to determine the amount of LC3BI/II protein #1 and #8 = standard marker ladder to determine molecular weight, shown in red. #2, #4 and #6 = probes of control cells (con) under basal conditions (#2), starvation (#4) and after Bafilomycin A1 treatment (#6) showing two distinct bands of the LC3BI and LC3BII protein, shown in red. #3, #5 and #7 = probes of NPC2-deficient cells (NP-C2) under same conditions a GAPDH is shown in green.
