# Supplementary material for: Pathophysiological In Vitro Profile of Neuronal Differentiated Cells Derived from Niemann-Pick Disease Type C2 Patient-Specific iPSCs Carrying the NPC2 Mutations c.58G>T/c.140G>T
Source: Int J Mol Sci. 2021 Apr 13;22(8):4009. doi: 10.3390/ijms22084009 (PMC8069078; doi:10.3390/ijms22084009)
Supplement: Supplementary file 1 [file ijms-22-04009-s001.zip › Supplementary Figure S4.pdf]

Supplementary Figure S4

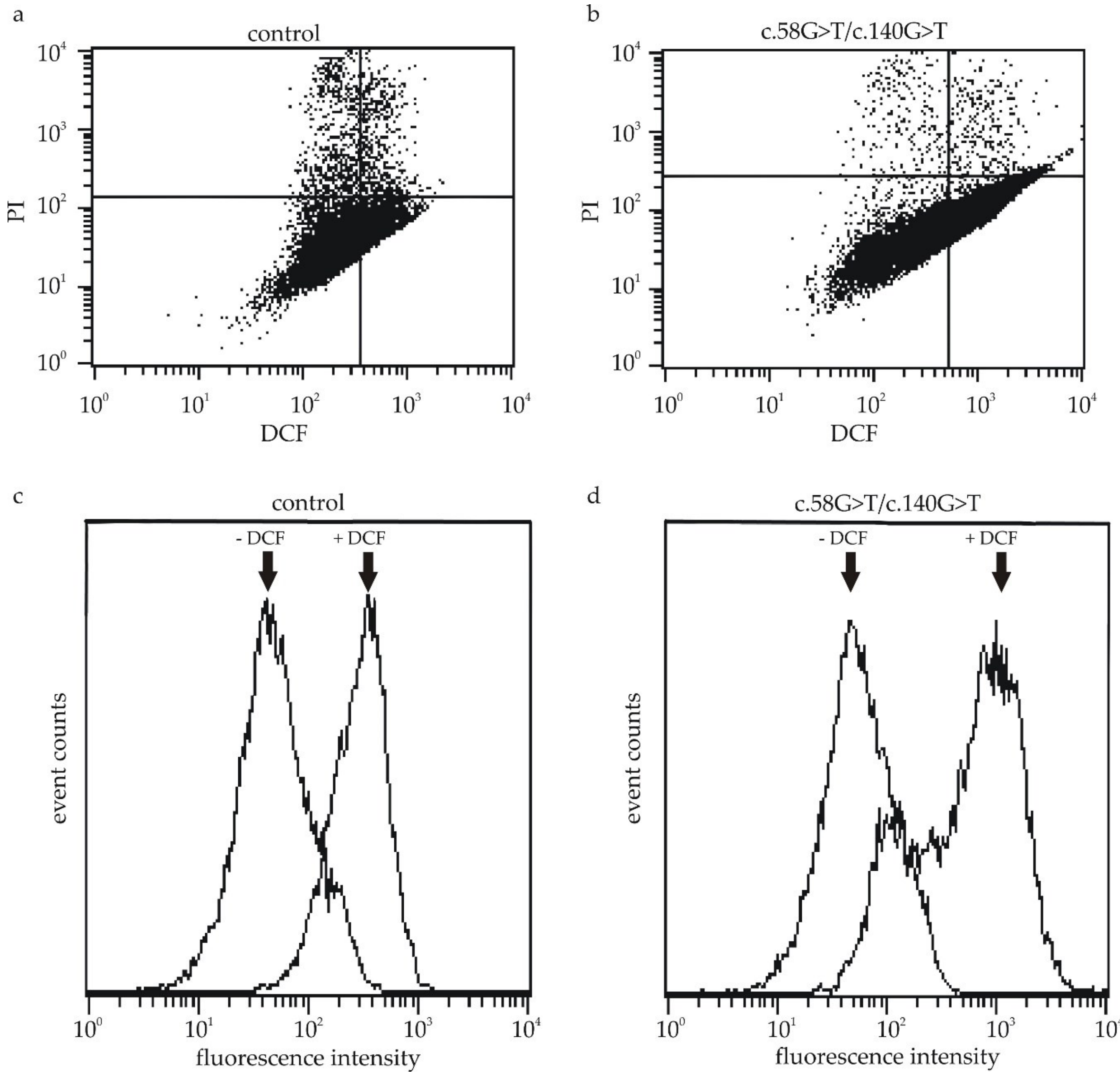

Supplementary Figure S4: Example of FACS analysis of ROS level by DCF fluorescence. (a) Dot plot of control cells and (b) of mutated cells simultaneously stained with propidium iodide (PI) and DCF. **Lower left quadrant**: double negative cells. **Lower right quadrant**: DCF positive cells and PI negative cells, reflecting cells suffering from oxidative stress (OS). **Upper left quadrant**: PI positive cells (necrotic/apoptotic cells). **Upper right quadrant**: PI and DCF positive cells. (c) Histogram of fluorescence distribution of control cells and (d) NPC2-deficient cells. **–DCF** = cells not treated with DCF, used as negative control to set the cut off for the fluorescence analysis. **+DCF** = cells treated with DCF. For both examples one can observe the right shift of the DCF fluorescence, indicating cells suffering from OS. Noticeably, the shift to a higher fluorescence intensity is more pronounced in the NPC2-deficient cell line.
