# Supplementary material for: Pathophysiological In Vitro Profile of Neuronal Differentiated Cells Derived from Niemann-Pick Disease Type C2 Patient-Specific iPSCs Carrying the NPC2 Mutations c.58G>T/c.140G>T
Source: Int J Mol Sci. 2021 Apr 13;22(8):4009. doi: 10.3390/ijms22084009 (PMC8069078; doi:10.3390/ijms22084009)
Supplement: Supplementary file 1 [file ijms-22-04009-s001.zip › Supplementary Table S1.pdf]

Supplementary Table S1:

**Antibodies used for Western blot**		
**Antibody**	**Dilution**	**Company**
catalase; rabbit IgG	1:1000	Cell Signaling Technology, Danvers, MA, USA
SOD1; rabbit IgG	1:10,000	Abcam, Cambridge, UK
SOD2; rabbit IgG	1:1000	Cell Signaling Technology, Danvers, MA, USA
GAPDH; mouse IgG	1:10,000	Abcam, Cambrigde, UK
β-Actin; mouse IgG	1:10,000	Sigma-Aldrich, St. Louis, USA
NPC2	1:1000	Abcam, Cambrigde, UK
LC3BI/II	1:1000	Cell Signaling Technology, Danvers, MA, USA
**Antibodies used for immunocytochemistry**		
**Antibody**	**Dilution**	**Company**
Sox2, rabbit IgG	1:200	Abcam, Cambrigde, UK
nestin, mouse IgG	1:100	R&D; Minneapolis, USA
Pax6, rabbit IgG	1:200	Abcam, Cambrigde, UK
βIII-tubulin, mouse IgG	1:100	Santa Cruz, Dallas, USA
GFAP, rabbit IgG	1:500	Sigma Aldrich, St. Louis, USA
Alexa Fluor 568; goat anti-mouse IgG	1:500	Invitrogen, Carlsbad, USA
Alexa Fluor 568; goat anti-rabbit IgG	1:500	Invitrogen, Carlsbad, USA
Alexa Fluor 488, goat anti-mouse IgG	1:500	Invitrogen, Carlsbad, USA

**Primer used for qRT-PCR**	
**Target**	**Forward/Reverse primer (5′- 3′)**
SOD1	AGGCCCCTTAACTCATCT / CTACAGGTACTTTAAAGCAACTCT
SOD2	GCACTAGCAGCATGTTGAGC / GCGTTGATGTGAGGTTCCAG
Catalase	TTTCCCAGGAAGATCCTGAC / ACCTTGGTGAGATCGAATGG
YWHAZ	GTCTGTAACTGAGCAAGGAGC / CTCTGCTTGTGAAGCATTGGG
